# Cyclin E1 overexpression sensitizes ovarian cancer cells to WEE1 and PLK1 inhibition

**DOI:** 10.1038/s41388-025-03312-4

**Published:** 2025-02-24

**Authors:** Qian Xi, Akiko Kunita, Miho Ogawa, Mirei Ka, Saki Tanimoto, Saki Tsuchimochi, Saeko Nagai, Asami Matsunaga, Tomohiko Fukuda, Kousuke Watanabe, Kenbun Sone, Aya Shinozaki-Ushiku, Kei Kawana, Tetsuo Ushiku, Yutaka Osuga, Kazuhiro Katayama, Hidenori Kage, Katsutoshi Oda

**Affiliations:** 1 Division of Integrative Genomics, Graduate School of Medicine, The University of Tokyo, Tokyo, Japan; 2 Next-Generation Precision Medicine Development Laboratory, Graduate School of Medicine, The University of Tokyo, Tokyo, Japan; 3 Department of Pathology, Graduate School of Medicine, The University of Tokyo, Tokyo, Japan; 4 Department of Obstetrics and Gynecology, Graduate School of Medicine, The University of Tokyo, Tokyo, Japan; 5 Department of Obstetrics and Gynecology, Nihon University School of Medicine, Tokyo, Japan; 6 Laboratory of Molecular Targeted Therapeutics, School of Pharmacy, Nihon University, Chiba, Japan; 7 Department of Respiratory Medicine, Graduate School of Medicine, The University of Tokyo, Tokyo, Japan

**Keywords:** Targeted therapies, Ovarian cancer

## Abstract

Cyclin E1 *(CCNE1)* amplification is associated with poor prognosis of ovarian carcinomas across histological subtypes. Inhibitors targeting PLK1 or WEE1 are emerging as promising therapeutic agents for cancer treatment that disrupt the critical G2/M checkpoint, leading to cancer cell death. However, biomarkers that predict the response to these inhibitors are not well defined. Here, we evaluated the efficacy of the PLK1 inhibitor, volasertib, and the WEE1 inhibitor, adavosertib, along with the biomarker potential of cyclin E1 in ovarian cancer cells. Both inhibitors suppressed the proliferation of cyclin E1-overexpressing cells to a greater extent than that of cells exhibiting low cyclin E1 expression. *TP53* silencing did not increase the sensitivity to these inhibitors. In cyclin E1-overexpressing cells, PLK1 inhibition reduced the proportion of cells in the G1 phase and increased those in the G2/M and sub-G1 phases. WEE1 inhibition reduced G1 phase cells without a clear peak in the S-G2/M phase and increased the sub-G1 phase cells. Both inhibitors suppressed the growth of cyclin E1-overexpressing tumors in vivo. Taken together, cyclin E1 overexpression, regardless of *TP53* status, may serve as a predictive biomarker for the efficacy of these inhibitors, offering potential personalized treatment strategies for ovarian cancer.

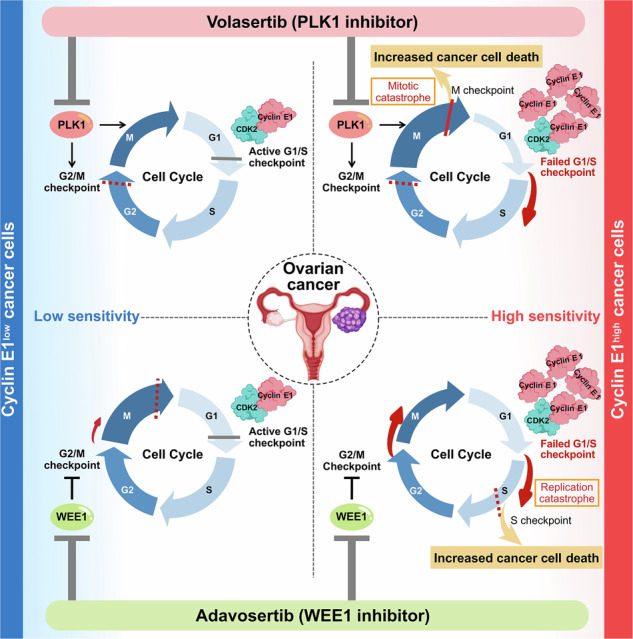

## Introduction

Epithelial ovarian cancer is a gynecological malignancy with a high mortality rate. There are multiple histological types, among which high-grade serous ovarian carcinoma (HGSOC) is the most prevalent, accounting for ~70% of deaths due to ovarian cancer [1], followed by endometrioid ovarian carcinoma (EOC) and clear cell ovarian carcinoma (CCOC) [2]. The prognosis of patients with platinum-resistant ovarian cancer is poor, highlighting the substantial unmet need for new treatment options [3]. *CCNE1* overexpression is a marker of homologous recombination (HR) proficiency and platinum resistance [4–6]. While CCOC accounts for only 4.8% of ovarian cancer cases in the United States, its frequency is higher in Asia, especially in Japan, accounting for ~25% [7]. CCOC tumors present unique therapeutic challenges, as they are generally HR-proficient with low platinum sensitivity [8, 9].

Cyclin E1, encoded by *CCNE1*, is crucial for the G1 to S phase cell cycle transition and is frequently overexpressed in ovarian cancers, particularly in HGSOC and CCOC. *CCNE1* amplification has been reported in ~20% of HGSOC cases [10], with overexpression and copy number gain observed in 23.3% and 14.8% of CCOC cases, respectively [11]. Amplification or overexpression of *CCNE1* is associated with chemoresistance and poor prognosis in ovarian cancer [11–15]. Cyclin E1 is overexpressed in 30% of established ovarian cancer cell lines [16]. Cyclin E1 overexpression exacerbates replication stress and is dependent on cell cycle checkpoints [17], highlighting the need for novel therapeutic strategies. The potential of Polo-like kinase 1 (PLK1) and WEE1 as therapeutic targets in this context is increasingly being explored.

PLK1 is a serine/threonine kinase essential for DNA repair, centrosome maturation, spindle assembly, and proper progression of mitosis [18]. Its overexpression in ovarian cancer has been implicated in chemoresistance, poor prognosis, and reduced survival [18–21]. Preclinical and early clinical trials have revealed pronounced anti-tumor activity of PLK1 inhibitors in ovarian cancer [22–24]. However, insights into the efficacy of PLK1 inhibitors as monotherapy in ovarian carcinomas (mainly HGSOC) remain limited [25–27]. The combination of PLK1 inhibitors with other agents, including PARP inhibitors and chemotherapeutic drugs, is complicated by concerns regarding dose optimization and adverse reactions [28–30]. The role of *TP53* mutations as biomarkers for the efficacy of PLK1 inhibitors in cancer is controversial. While some studies have indicated that *TP53* status can influence the sensitivity of cancer cells to PLK1 inhibition [31], other reports suggest no direct association between *TP53* loss and PLK1 inhibitor sensitivity [32].

WEE1, a tyrosine kinase essential for the G2-M transition, maintains cell viability in the presence of DNA damage [33–35]. Its overexpression is observed in several malignant conditions, including ovarian cancer [36]. Inhibition of WEE1 increases genomic instability and sensitizes cells to DNA-damaging agents. Cells with an impaired G1/S checkpoint (i.e., *TP53* mutations) show a high dependency on the G2/M checkpoint [37]. WEE1 inhibitors can induce premature mitosis and subsequent apoptosis as a result of mitotic entry with unrepaired DNA damage [38]. A phase II study of the WEE1 inhibitor, adavosertib, (AZD1775) showed an objective response rate of 29.4% in recurrent uterine serous carcinoma, with *TP53* mutations in over 90% of cases and *CCNE1* alterations in 26% of cases [38, 39]. Adavosertib has shown efficacy, either as a single agent or in combination with carboplatin, in patients with platinum-resistant ovarian cancer in Phase II clinical trials [40–42]. However, *TP53* mutations are not the best predictive biomarkers, as determined in preclinical and clinical studies [43–45]. Trials are underway for assessing WEE1 inhibitors in combination with other agents, including ATR and PARP inhibitors as well as anti-PD-L1 immunotherapy [44]. Although adavosertib has been suggested to also target PLK1 [46], it is questionable whether PLK1 inhibition occurs at clinically relevant concentrations, and its classification as a WEE1/PLK1 dual inhibitor is not as well-established [47].

As cyclin E1 is associated with CDK2, and together they promote G1/S transition and DNA replication, cyclin E1 overexpression is a candidate predictive biomarker of PLK1 and WEE1 inhibitor sensitivity. Although previous studies on these inhibitors have mainly focused on cells with *TP53* mutations [42, 48], we hypothesized that cyclin E1 overexpression is a potential predictive biomarker of PLK1 and WEE1 inhibitors, regardless of the *TP53* status. In the present study, we determined the efficacy of PLK1 and WEE1 inhibitors in ovarian cancer cells overexpressing cyclin E1.

## Results

### The sensitivity of PLK1 or WEE1 inhibitors correlates with cyclin E1 expression, rather than *CCNE1* amplification

**Fig. 1:**
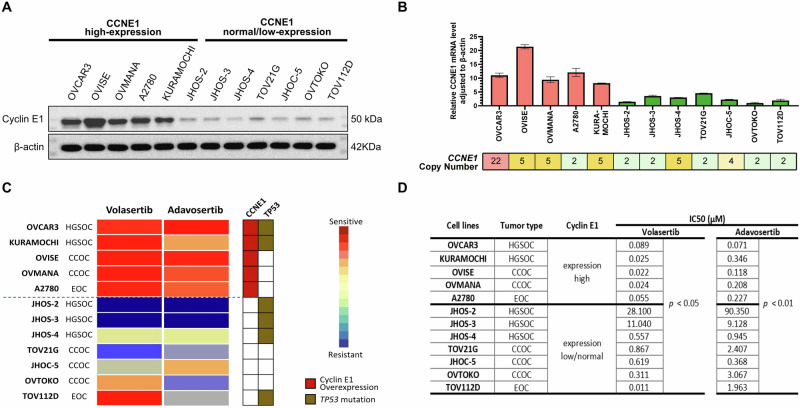
The sensitivity of PLK1 or WEE1 inhibitors correlates with cyclin E1 expression, rather than *CCNE1* amplification in ovarian cancer cell lines. **A** Protein expression of Cyclin E1 and β-actin, as determined via western blotting. **B** mRNA expression levels of *CCNE1* and its expression relative to β-actin, as determined via RT-qPCR. *CCNE1* copy numbers were obtained from the DepMap portal and Cell Model Passports databases. **C** Heatmap showing drug sensitivity of ovarian cancer cell lines to volasertib and adavosertib, assessed via cell viability assays. The status of *CCNE1* and *TP53* is indicated. Drug sensitivity is measured based on adjusted IC_50_ values. **D** IC_50_ values of the corresponding cell lines, as presented in (**C**), with a comparison of IC_50_ values between the *CCNE1* high and low/normal expression groups. HGSOC high-grade serous ovarian carcinoma, CCOC clear cell ovarian carcinoma, EOC endometrioid ovarian carcinoma.

First, we validated the expression levels of cyclin E1 in 12 ovarian cancer cell lines representing different histological types. Both protein (cyclin E1) and mRNA (*CCNE1*) levels were concordantly high in OVCAR3, OVISE, OVMANA, A2780, and KURAMOCHI cells while low in JHOS-2, JHOS-3, JHOS-4, TOV21G, JHOC-5, OVTOKO, and TOV112D cells (Fig. 1A, B). These data suggest that the *CCNE1* mRNA and protein expression correlate well, with high cyclin E1 expression observed across various histological types. However, the *CCNE1* copy number did not consistently correlate with *CCNE1* expression (Fig. 1B).

We performed a drug sensitivity assay across the 12 ovarian cancer cell lines to explore the therapeutic potential of PLK1 inhibitor volasertib and WEE1 inhibitor adavosertib in ovarian cancer. The IC_50_ values of volasertib and adavosertib were significantly lower in cells with high *CCNE1* expression than in those with low/normal *CCNE1* expression (Fig. 1C, D). The IC_50_ values for volasertib ranged from 0.022 to 0.089 μM in cells with elevated *CCNE1* expression compared to values ranging from 0.311 to 28.1 μM in six out of the seven cell lines with low/normal *CCNE1* expression, except for TOV112D. Likewise, the IC_50_ values for adavosertib ranged from 0.071 to 0.346 μM in cells with elevated *CCNE1* expression and from 0.368 to 90.4 μM in cells with low/normal *CCNE1* expression (Fig. 1C, D). The *TP53* status (or histology) was not significantly associated with sensitivity to volasertib and adavosertib (Fig. 1C). These data suggest that *TP53* mutation alone may not be a reliable biomarker, and cyclin E1 overexpression may be a more suitable biomarker for both PLK1 and WEE1 inhibitor sensitivity in ovarian cancer cells.

We compared the phosphorylation levels of key cell cycle regulators between Cyclin E1-overexpressing cells and Cyclin E1-low/normal-expressing cells by western blotting. The phosphorylation levels of CDK2 at Thr160 were significantly higher in Cyclin E1-overexpressing cells, indicating that the Cyclin E1-CDK2 complex was highly activated in these cells, thereby driving abnormal cell cycle progression (Fig. S1). Additionally, the elevated expression and phosphorylation levels of Rb and CDK1 suggested that Cyclin E1 overexpression promoted deregulation across the entire cell cycle, although the differences between the two groups were not statistically significant (Fig. S1).

### *TP53* inactivation does not universally predict the response to PLK1 and WEE1 inhibitors

**Fig. 2:**
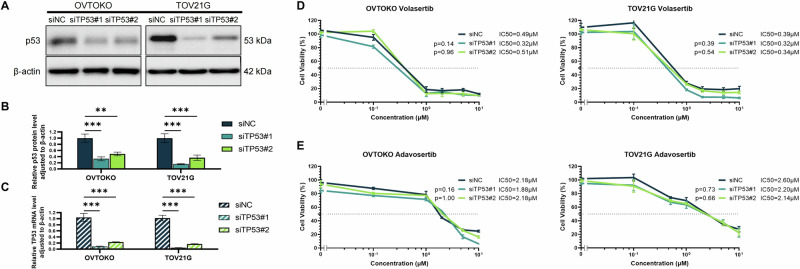
*TP53* inactivation is not a universal predictive biomarker of the response to PLK1 and WEE1 inhibitors. Validation of *TP53* knockdown efficiency with two types of siRNA specific to *TP53* based on TP53 protein levels (**A**, **B**) and mRNA expression (**C**) in OVTOKO and TOV21G cell lines. Analysis of cell viability in response to volasertib (**D**) or adavosertib (**E**) under *TP53* knockdown. Reported *p* values were determined via one-way ANOVA based on IC_50_ values obtained from multiple repeated experiments. Statistical significance is denoted as **p < 0.01 and ***p < 0.001.

To assess the role of *TP53* in PLK1 and WEE1 inhibitor sensitivity, we knocked down *TP53* in OVTOKO and TOV21G cells, which are *TP53* wild-type and have low cyclin E1 expression. We used two siRNAs specific to *TP53* (siTP53#1 and siTP53#2) and confirmed knockdown efficiency via western blotting and RT-qPCR (Fig. 2A–C). *TP53* knockdown did not significantly increase the sensitivity to volasertib or adavosertib in these two cell lines (Fig. 2D, E).

We evaluated the effect of *TP53* loss using two p53KO−/− cell lines (A549 and U2OS) (Fig. S2A). Neither volasertib nor adavosertib sensitivity was affected in either cell line (Fig. S2B–E). These findings support the notion that *TP53* loss alone is not a universal predictive biomarker of PLK1 and WEE1 inhibitor sensitivity.

### Cyclin E1 overexpression is a predictive biomarker of PLK1 and WEE1 inhibitor efficacy

**Fig. 3:**
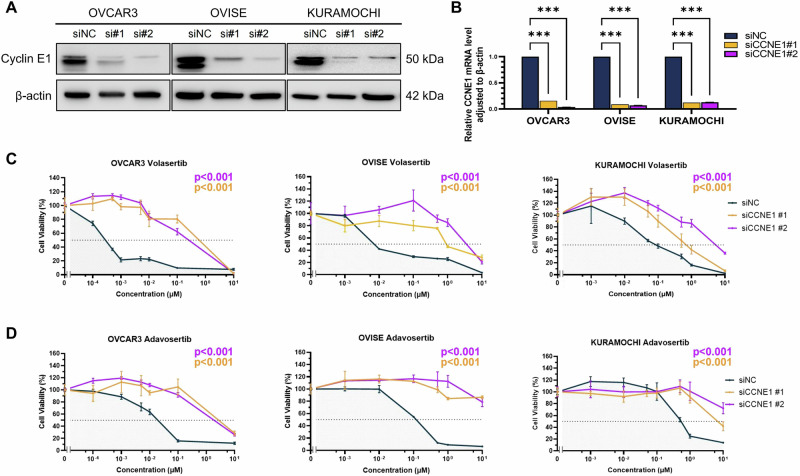
Cyclin E1 expression is a predictive biomarker for the efficacy of PLK1 and WEE1 inhibitors. Knockdown efficiency of *CCNE1* using siRNA (siCCNE1) was assessed based on protein (**A**) and mRNA (**B**) levels. Cell viability assays following *CCNE1* knockdown in OVCAR3, OVISE, and KURAMOCHI cells treated with PLK1 inhibitor volasertib (**C**) or WEE1 inhibitor adavosertib (**D**). The *p* values were determined via one-way ANOVA based on IC_50_ values obtained from multiple repeated experiments. Statistical significance is denoted as ***p < 0.001.

To assess the impact of cyclin E1 overexpression on the sensitivity to PLK1 and WEE1 inhibitors, we performed siRNA-mediated knockdown in cell lines with high cyclin E1 expression (OVCAR3, OVISE, and KURAMOCHI). Knockdown efficiency of both *CCNE1*-specific siRNAs (siCCNE1#1 and siCCNE1#2) was confirmed using western blotting and RT-qPCR (Fig. 3A, B). Cell viability assay revealed that, compared with the control siRNA, *CCNE1* knockdown significantly decreased volasertib and adavosertib sensitivity in these three cell lines (siNC) (*p* < 0.001, Fig. 3C, D). This finding underlines the significance of cyclin E1 as a predictive biomarker for the efficacy of both PLK1 and WEE1 inhibitors in ovarian cancer.

Next, we overexpressed Cyclin E1 in the Cyclin E1-low-expressing JHOC-5 cells using a doxycycline-inducible PiggyBac vector system. Cyclin E1 expression was upregulated by ~12-fold (Fig. S3A, B). This Cyclin E1 overexpression significantly increased the sensitivity of JHOC-5 cells to both volasertib and adavosertib, as demonstrated by a marked reduction in IC_50_ values compared to control cells (Fig. S3C). These results indicate that Cyclin E1 overexpression is a key determinant of drug sensitivity, which enhances the efficacy of both volasertib and adavosertib.

### Cyclin E1 overexpression sensitizes ovarian cancer cells to PLK1 and WEE1 inhibitors via cell cycle dysregulation

**Fig. 4:**
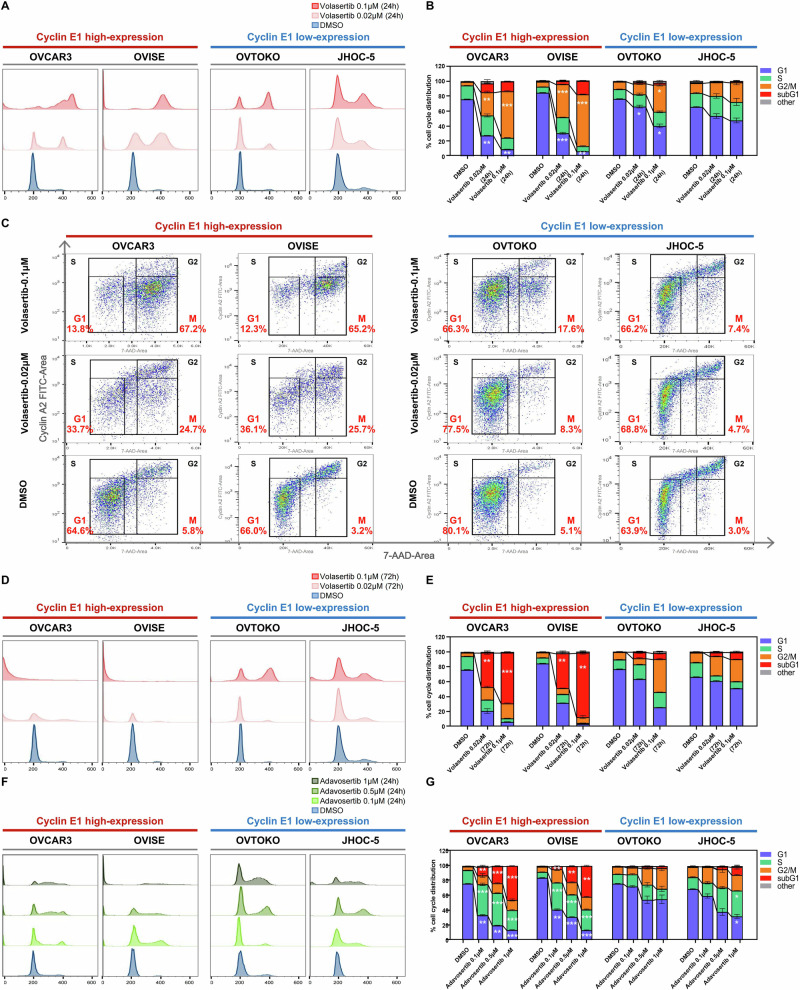
Cyclin E1 overexpression sensitizes ovarian cancer cells to PLK1 or WEE1 inhibitors by deregulating the cell cycle. **A**–**E** Cells were exposed to DMSO (control) or two concentrations of volasertib (0.02 and 0.1 μM) for 24 to 72 h in four ovarian cancer cell lines: cyclin E1-high (OVCAR3, OVISE) and cyclin E1-low (OVTOKO, JHOC-5). **A**, **B** Flow cytometry analysis and corresponding cell cycle phase distribution after 24 h of volasertib treatment, with statistical analysis (One-Way ANOVA). **C** Cyclin A2 and 7-AAD dual staining showing the proportion of cells in G1, S, M, and G2 phases (excluding sub-G1 and others). **D**, **E** Cells were treated with volasertib for 72 h. **F**, **G** Cells were exposed to DMSO (control) or three concentrations of adavosertib (0.1, 0.5, and 1 μM) for 24 h in the four cell lines. All comparisons were made between DMSO- and volasertib- or adavosertib-treated groups. Statistical significance was assessed by One-Way ANOVA (*p < 0.05; **p < 0.01; ***p < 0.001).

Given the role of cyclin E1 in the G1/S transition as well as the involvement of PLK1 and WEE1 in the G2/M checkpoint, we evaluated the effect of both volasertib and adavosertib on cell cycle regulation using flow cytometry (Fig. 4A–G). Twenty-four-hour treatment with volasertib (0.02 or 0.1 μM) significantly reduced the proportion of cells in the G1 phase while increasing those in the G2/M phase in two cell lines with high cyclin E1 expression (OVCAR3 and OVISE) (Fig. 4A, B). G2 and M phases were further distinguished by co-staining with cyclin A2, which revealed that volasertib predominantly increased the M phase in the two cell lines with high cyclin E1 expression (Fig. 4C). This M phase arrest was significantly weaker in the two cell lines with low cyclin E1 expression (OVTOKO and JHOC-5). Extending volasertib treatment for 72 h in cyclin E1-overexpressing cells markedly increased the sub-G1 phase population, indicating cell death (Fig. 4D, E). The ratio of cells in the sub-G1 phase was much lower in cells with low cyclin E1 expression.

Similarly, WEE1 inhibition by adavosertib (0.1, 0.5, or 1 μM) markedly decreased the G1 ratio, especially in high cyclin E1-expressing cells, 24 h after treatment (Fig. 4F, G). Of note, adavosertib remarkably increased the S ratio in high cyclin E1-expressing cells (Fig. 4G). The G2/M ratio in these cells was less affected by adavosertib treatment (Fig. 4G). Furthermore, the proportion of sub-G1 phase cells was significantly increased by adavosertib treatment, especially in high cyclin E1-expressing cells 24 h after treatment, in a dose-dependent manner (Fig. 4G).

### PLK1 and WEE1 inhibitors induce apoptosis in cyclin E1-overexpressing ovarian cancer cells

**Fig. 5:**
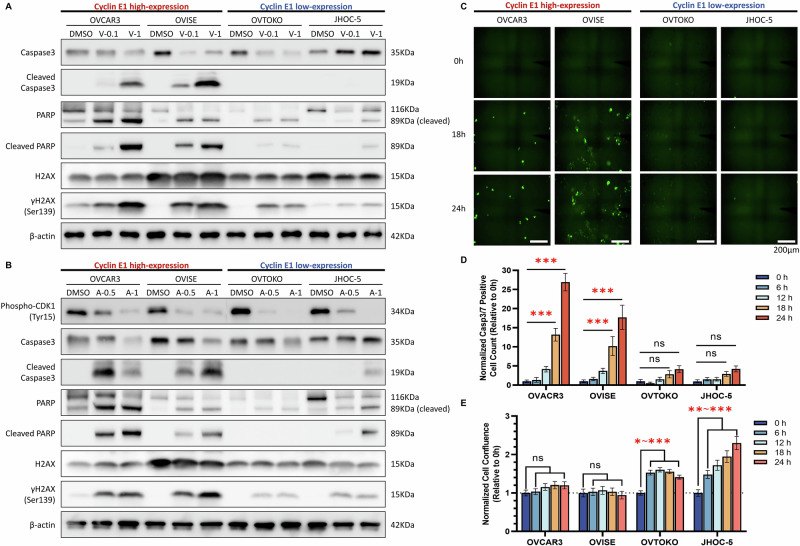
PLK1 and WEE1 inhibitors induce apoptosis in cyclin E1-overexpressing ovarian cancer cells. **A** Protein levels of apoptosis-related proteins (cleaved caspase-3 and cleaved PARP) and γH2AX (indicative of DNA double-strand breaks) at 72 h after treatment of cyclin E1-high (OVCAR3, OVISE) and cyclin E1-low cells (OVTOKO, JHOC-5) with 0.1 and 1 µM volasertib evaluated using western blotting. **B** Protein levels of phospho-CDK1 (Tyr15), apoptosis-related proteins, and γH2AX at 72 h after treatment with 0.5 and 1 µM adavosertib in the four cell lines described in (**A**). V-0.1, volasertib 0.1 µM; V-1, volasertib 1 µM; A-0.5, adavosertib 0.5 µM; A-1, adavosertib 1 µM. **C**–**E** Time-lapse microscopy analysis of apoptosis in Cyclin E1-high and Cyclin E1-low expressing cells. **C** Representative images of Caspase-3/7 Green fluorescence, indicating apoptotic cells, captured at 0, 18, and 24 h. **D** Normalized Caspase-3/7 positive cell counts expressed relative to 0 h. **E** Normalized cell confluence, expressed relative to 0 h. Data are shown as mean ± SEM. Statistical significance is indicated as *p < 0.05, **p < 0.01, ***p < 0.001, and ns (not significant).

To elucidate the impact of PLK1 and WEE1 inhibitors on apoptosis, we evaluated protein levels associated with apoptosis and DNA double-strand breaks. Both cleaved caspase-3 and cleaved PARP were induced by volasertib and adavosertib treatment, specifically in cyclin E1-overexpressing cells (Fig. 5A, B). Volasertib and adavosertib treatment also increased the levels of γH2AX (histone H2AX phosphorylated at serine 139), a marker of DNA double-strand breaks, in cyclin E1-overexpressing cells (Fig. 5A, B). These findings are consistent with the increase in sub-G1 cells, suggesting that apoptosis was induced by these inhibitors (Fig. 4E, G). We further confirmed that adavosertib downregulated the phosphorylation levels of CDK1 (Tyr15), a downstream target of WEE1, regardless of the Cyclin E1 expression status (Fig. 5B).

Furthermore, the time-lapse microscopy experiment revealed a time-dependent increase in apoptosis (Casp3/7 positive cells) following adavosertib treatment, specifically in Cyclin E1-overexpressing cells. Apoptotic cells, marked by green fluorescence, were evident after 18 h of treatment (Fig. 5C, D). Normalized cell confluence analysis revealed no significant cell growth over 24 h in Cyclin E1-overexpressing cells (Fig. 5E). In contrast, Cyclin E1-low-expressing cells showed minimal or no detectable apoptosis throughout the observation period of cell growth (Fig. 5C–E). These findings suggest that cell cycle dysregulation by adavosertib could induce apoptosis in Cyclin E1-overexpressing cells.

### Combination of PLK1 and WEE1 inhibitors induces additive anti-proliferative effect in certain cyclin E1-overexpressing ovarian cancer cells

We evaluated the effects of combination therapy with volasertib and adavosertib compared to single-agent treatments in Cyclin E1-overexpressing cell lines. Isobologram analysis revealed additive effects in OVISE and KURAMOCHI cells, while antagonistic effects were observed in OVCAR3 cells (Fig. S4). These findings indicate that the efficacy of combination therapy is context-dependent and may be limited, but can offer therapeutic potential in specific settings.

### Cyclin E1 expression is a predictive biomarker of PLK1 or WEE1 inhibitor sensitivity in vivo

**Fig. 6:**
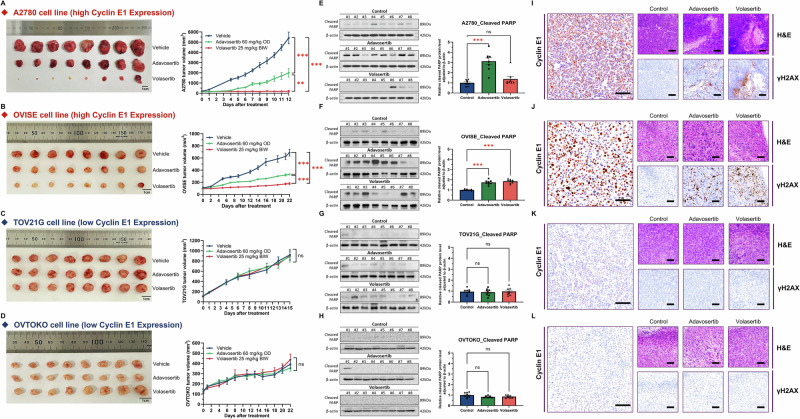
Cyclin E1 expression is a predictive biomarker of the response to PLK1 or WEE1 inhibitors in vivo. **A**–**D** Mouse xenograft experiments in 6-week-old female BALB/cSlc-nu/nu mice inoculated with four ovarian cancer cells. Mice were treated with vehicle, volasertib BIW (twice weekly) at 25 mg/kg, or adavosertib OD (once daily) at 60 mg/kg via oral administration for 12–22 days (*n* = 8 per group). The figure includes photos of resected tumors and graphs showing tumor volume. A two-way ANOVA with Tukey’s multiple comparisons test was used to compare various experimental arms with the vehicle arm. **E**–**H** Analyses of cleaved PARP levels via western blotting. One-way ANOVA or the Kruskal–Wallis test was used for analysis. **I**–**L** Representative H&E staining and detection of cyclin E1 and γH2AX (Scale bar, 100 μm) by immunohistochemistry. Cyclin E1-high cells: **A** A2780, **B** OVISE, and cyclin E1-low cells: **C** TOV21G and **D** OVTOKO. Statistical significance is denoted as ns, non-significant; **p < 0.01; and ***p < 0.001.

We evaluated the anti-tumor effects of PLK1 and WEE1 inhibitors in vivo by using mouse xenograft models with high (A2780 and OVISE) and low (TOV21G and OVTOKO) cyclin E1-expressing cells. Oral administration of volasertib (60 mg/kg daily) or adavosertib (25 mg/kg twice weekly) for 12–22 days caused tumor regression in A2780 and OVISE tumors (Fig. 6A, B). Conversely, none of the inhibitors exhibited a significant cytostatic effect in TOV21G and OVTOKO tumors (Fig. 6C, D). No apparent side effects, such as body weight loss, were observed in mice treated with volasertib or adavosertib. Additionally, cleaved PARP was upregulated by both treatments in the high cyclin E1 expression model (*p* < 0.001; Fig. 6E, F), but not in the low cyclin E1-expression model (Fig. 6G, H).

Further evaluation of drug effects via immunohistochemistry (IHC) of the xenograft revealed that both volasertib and adavosertib treatment increased γH2AX levels in the high cyclin E1 expression model (A2780 and OVISE, Fig. 6I, J), but not in the low cyclin E1 expression model (TOV21G and OVTOKO, Fig. 6K, L). Cyclin E1 expression remained high in xenografts derived from A2780 and OVISE cells (Fig. 6I, J).

## Discussion

Our findings demonstrated that (i) both the PLK1 inhibitor, volasertib, and WEE1 inhibitor, adavosertib, exhibited anti-tumor effects in ovarian cancer cells of various histological subtypes both in vitro and in vivo. (ii) Cyclin E1 overexpression was a significant predictor of sensitivity to both inhibitors, and (iii) *TP53* inactivation itself was not associated with sensitivity to these inhibitors.

Both volasertib and adavosertib have been examined in various clinical trials [39, 41, 49–51]. However, they have not been tested concomitantly, and predictive biomarkers for their effectiveness have yet to be identified. Our data clearly showed that ovarian cancer cell lines sensitive to these inhibitors overlapped significantly and that this sensitivity was strongly associated with cyclin E1 overexpression. These findings highlight the similarities between the anti-tumor effects of PLK1 and WEE1 inhibitors.

**Fig. 7:**
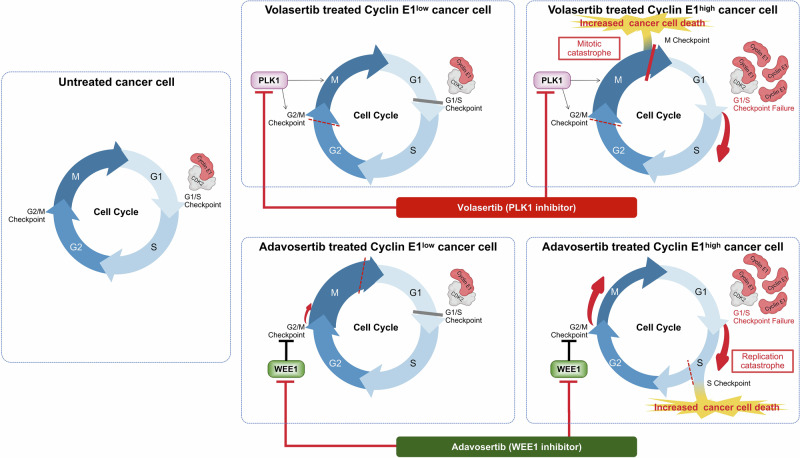
PLK1 and WEE1 inhibitors induce synthetic lethality in cyclin E1-overexpressing cells through different mechanisms of action. The diagram illustrates the mechanisms and differential responses to treatment with volasertib and adavosertib of high cyclin E1-expressing cells versus low cyclin E1-expressing cells. The figure highlights how these drugs affect cell cycle progression and apoptosis in cells with varying levels of cyclin E1. This model proposes that synthetic lethality with a PLK1 inhibitor takes advantage of the arrested mitotic (spindle) assembly checkpoint and mitotic catastrophe (during the M phase), while lethality induced by a WEE1 inhibitor depends on the persistence of unrepaired DNA damage and replication catastrophe (during the S phase).

As both PLK1 and WEE1 inhibitors disrupt the G2/M checkpoint and DNA damage response, it is rather plausible that these inhibitors exhibit similar anti-tumor activities with common predictive biomarkers, such as cyclin E1 overexpression. Cell cycle dynamics highlight the differential responses of cells to volasertib and adavosertib based on high and low cyclin E1 expression (Fig. 7). This diagram integrates the findings of our study with the established roles of PLK1, WEE1, and cyclin E1 in cell cycle regulation. The cyclin E1/ CDK2 complex is a key driver of the G1/S checkpoint. When the expression of cyclin E1 is low, and the cyclin E1/CDK2 complex is under normal cell cycle control, cells remain in the G1 phase to repair DNA damage. Although either a PLK1 inhibitor or a WEE1 inhibitor may promote evasion of the G2/M checkpoint in this context, the G1/S checkpoint acts as a safeguard, preventing cell cycle progression in cells with low cyclin E1 levels. However, in cells overexpressing cyclin E1, the active cyclin E1/CDK2 complex drives disruption of the G1/S checkpoint, as observed following treatment with the PLK1 and WEE1 inhibitors (Fig. 7). The differences in mode of action between the PLK1 and WEE1 inhibitors lies in the specific mechanism through which they impair the G2/M checkpoint. The PLK1 inhibitor strongly blocks the transition from G2 to M, preventing cells from halting in the G1 phase when cyclin E1 is overexpressed [52]. This leads to accumulated DNA damage, an arrested mitotic (spindle) assembly checkpoint (i.e., arrest in M checkpoint), and mitotic catastrophe, ultimately causing cell death (Fig. 7). Indeed, our cell cycle analysis following volasertib treatment showed that G2/M arrest was extensively induced in cells with overexpressing cyclin E1. In addition, the reduction in the G1 phase, accompanied by an increase in the sub-G1 population, was more severe in cyclin E1-overexpressing cells. WEE1 inhibition disrupts the G2/M checkpoint, driving G2 to M phase transition [53, 54], leading to an uncontrolled cell cycle under cyclin E1 overexpression. With no blockade at the G1/S or G2/M checkpoints, unrepaired DNA damage accumulates, leading to replication catastrophe during the S phase (i.e., arrest via the intra-S checkpoint), ultimately causing cell death (Fig. 7). Although mitotic catastrophe might have contributed partially, our cell cycle analysis following adavosertib treatment showed an accumulation of cells in the S phase, rather than the G2/M phase, particularly among cyclin E1-overexpressing cells, even at a low dose (0.1 and 0.5 μM). Additionally, a more pronounced reduction in the G1 and an increase in the sub-G1 population were noted in cyclin E1-overexpressing cells, in a dose-dependent manner. Western blotting showed that both inhibitors increased the levels of apoptosis markers and γH2AX in cells with high cyclin E1 expression, highlighting their cytotoxic effects and suggesting an interplay between cyclin E1, the DNA damage response, and apoptosis.

Notably, *TP53* inactivation did not significantly affect the sensitivity to these inhibitors in our study. The correlation between *TP53* status and sensitivity to PLK1 inhibitors is not well-established [55, 56]. Volasertib did not increase apoptotic cell death in A549 lung cancer cells following *TP53* inactivation [57]. The correlation between *TP53* mutations and sensitivity to WEE1 inhibitors has been extensively studied [58]. WEE1 inhibitors have shown potential efficacy in *TP53*-mutated cancer cells when combined with conventional chemotherapy and ionizing radiation [59, 60]. Adavosertib monotherapy has been investigated in clinical trials for patients with ovarian cancer, particularly those with *TP53* mutations, in addition to patients with uterine serous carcinoma [39, 41, 42, 61]. Gemcitabine combined with adavosertib has been evaluated for patients with platinum-resistant or refractory high-grade serous ovarian cancer [40]. Therefore, clinical trials for ovarian cancer with wild-type *TP53* are limited. Recently, a phase II clinical trial of adavosertib showed an overall response rate of 27% with a partial response in eight patients with refractory solid tumors harboring *CCNE1* amplification, including ovarian cancer [62]. In this trial, 90% of patients harbored *TP53* mutations with *CCNE1* amplification. Our data provides valuable evidence that *CCNE1* amplification, rather than *TP53* mutations, may serve as a suitable biomarker for WEE1 inhibitors. Our findings highlight the potential therapeutic effects of PLK1 inhibitors in ovarian cancer with cyclin E1 overexpression. Given that *TP53* mutations are closely associated with histological subtypes (e.g., nearly all in high-grade serous carcinomas but rare in other histological types) [10, 63], both PLK1 and WEE1 inhibitors could be promising therapeutic candidates with selective biomarkers in precision medicine for various types of ovarian cancer.

When considering cyclin E1 as a biomarker, it is crucial to determine whether cyclin E1 protein levels or *CCNE1* copy number amplification should be considered. Our study indicated a discordance between cyclin E1 expression and *CCNE1* copy number amplification in ovarian cancer cells, consistent with previous reports [64]. Furthermore, while high-level *CCNE1* amplification (>8 copies) significantly correlates with cyclin E1 protein overexpression in HGSOC, such amplification is found in only 8.6% of HGSOC cases. Additionally, 21.1% of HGSOC cases exhibited cyclin E1 overexpression without high-level *CCNE1* amplification [65]. Since cyclin E1 overexpression can be evaluated via IHC, high cyclin E1 protein expression may be a more suitable biomarker for clinical settings.

Our study has limitations that warrant further consideration. First, the threshold for *CCNE1* overexpression as a biomarker could not be precisely determined. Companion diagnostics, such as the detection of high cyclin E1 levels via IHC (and/or *CCNE1* copy number amplification), should be developed. Second, whether combining these inhibitors with other drugs, such as targeted therapies or chemotherapy, may enhance their anti-tumor effects, is unclear. Third, our analysis was limited to ovarian cancer cells, and the significance of cyclin E1 overexpression as a biomarker for PLK1 and WEE1 inhibitor sensitivity should be investigated in other cancer types.

In conclusion, we demonstrated the critical role of cyclin E1 in the response to PLK1 and WEE1 inhibitors in ovarian cancer. Leveraging synthetic lethality by targeting cyclin E1 overexpression with either PLK1 or WEE1 inhibitors may offer a viable therapeutic strategy for ovarian cancer, irrespective of the histological type.

## Materials and methods

### Cell lines and primary cells

TOV21G, TOV112D, and U2OS cell lines were purchased from the American Type Culture Collection (ATCC, Manassas, VA, USA). OVCAR3, JHOC-5, JHOS-2, JHOS-3, JHOS-4, and A549 cells were purchased from the RIKEN CELL BANK (Tsukuba, Japan). KURAMOCHI, OVMANA, OVISE, and OVTOKO cell lines were purchased from the Japanese Collection of Research Bioresources Cell Bank (JCRB, Osaka, Japan). A2780 was obtained from ECACC (Salisbury, United Kingdom). We employed 12 cell lines of different histological type, *TP53* status, and cyclin E1 expression, including five HGSOCs, five CCOCs, and two EOCs. TOV21G, TOV112D, A2780, OVCAR3, KURAMOCHI, OVMANA, OVISE, and OVTOKO cells were cultured in RPMI 1640 medium (Thermo Fisher Scientific) supplemented with 10% fetal bovine serum (FBS; Sigma-Aldrich) and 1% antibiotics (penicillin) (Gibco; Thermo Fisher Scientific, Waltham, MA). JHOC-5, JHOS-2, JHOS-3, and JHOS-4 cells were cultured in DMEM/HamF12 medium (Thermo Fisher Scientific) with 10% FBS, 0.1 mM NEAA (Thermo Fisher Scientific), and 1% antibiotics. All cell lines were cultured in a humidified incubator at 37 °C with 5% CO_2_ and were not passaged more than 15 times.

A549 sgRNA-NC, A549 p53KO−/−, U2OS sgRNA-NC, and U2OS p53KO−/− cell lines, which were generated using CRISPR/Cas9 technology, were a kind gift from Dr. Koichi Matsuda (Graduate School of Frontier Sciences, The University of Tokyo). A549 cells were cultured in RPMI 1640 medium supplemented with 10% FBS and 1% antibiotics. U2OS cells were cultured in McCoy’s 5A medium (Gibco) supplemented with 2 mM L-glutamine (Gibco), 10% FBS, and 1% antibiotics.

### Mouse models

A2780, OVISE, OVTOKO, and TOV21G tumor cells (1 × 10^7^ cells in 200 µL PBS) were subcutaneously injected into 6-week-old female BALB/cSlc-nu/nu mice (Japan SLC). Xenograft experiments were conducted under specific pathogen-free (SPF) conditions in an SPF animal facility, in compliance with institutional and international guidelines. Mice were housed in individually ventilated metal cages (three mice per cage) under a 12-h light/dark cycle, at a controlled temperature of 22 ± 2 °C and a humidity of 50 ± 10%. After the tumors reached a size of 100–200 mm^3^, the mice were treated with vehicle, volasertib, BIW (twice weekly) at 25 mg/kg, or adavosertib OD (once daily) at 60 mg/kg for 12–22 days. Mice were randomly assigned into treatment groups (vehicle, volasertib, and adavosertib) with eight mice per group to ensure unbiased allocation. Adavosertib was freshly prepared in 0.5% methylcellulose (FUJIFILM Wako, Osaka, Japan), and volasertib was prepared in corn oil (FUJIFILM Wako). Both formulations are prepared weekly to ensure stability and cover the dosage for the following week. Tumor growth was measured three times weekly, and volumes were calculated using the following formula: (4π/3) × (width/2)^2^ × (length/2). No blinding was performed during the animal study. However, tumor measurements and analyses were conducted using objective methods to minimize potential bias. After sacrificing the mice, the resected tumors were divided into two parts: one part was frozen for protein extraction, and the other was fixed in 10% Formalin Neutral Buffer Solution (FUJIFILM Wako; 062-01661) for immunohistochemical analysis.

### *CCNE1* copy number and *TP53* status in ovarian cancer cell lines

*CCNE1* copy number for each cell line was obtained from the DepMap portal and Cell Model Passports databases, with expression data referred from previous research [64]. The annotation of *TP53* mutations was derived from the COSMIC (Catalogue of Somatic Mutations in Cancer) and ClinVar databases.

### RNA extraction and quantitative real-time polymerase chain reaction

Total RNA was extracted from cells using the RNeasy Mini Kit (Qiagen, Valencia, CA, USA). Complementary DNA was prepared from total RNA using ReverTra Ace qPCR Master Mix with gDNA Remover (TOYOBO, Osaka, Japan). Quantitative real-time polymerase chain reaction (qRT-PCR) was conducted using a One-Step SYBR Prime Script RT-PCR Kit (TaKaRa Bio, Tokyo, Japan) on a Light Cycler instrument (Roche Diagnostics, Indianapolis, IN, USA) and a QuantStudio 1 Real-Time PCR System (Thermo Fisher Scientific; A40425). Relative gene expression was analyzed using the 2^−∆∆Ct^ method. All experiments were performed in triplicates.

### Protein extraction and western blotting

Proteins were extracted from cells using RIPA buffer (FUJIFILM Wako). Cells were treated with volasertib or adavosertib for 72 h for apoptosis and DNA damage-related assays, and cells were transfected with siCCNE1 for 48 h. The samples were subjected to intermittent ultrasonication for 10 min for disruption. Proteins were denatured after extraction by boiling in 4× Laemmli sample buffer (Bio-Rad, Hercules, CA, USA; #1610747) at 95 °C for 5 min. Subsequently, the proteins were separated using 4–15% or Any kD™ Mini-PROTEAN® TGX™ Precast Protein Gels (Bio-Rad; #4561084, #4569033), and the separated proteins were transferred onto Trans-Blot® Turbo™ Mini PVDF Transfer Packs (Bio-Rad; 1704156). The membranes were incubated with primary antibodies in the following dilutions: Cyclin E1 (HE12) (Santa Cruz Biotechnology, #sc-247), Caspase-3 (Cell Signaling Technology [CST], #9662), Cleaved Caspase-3 (Asp175) (5A1E) (CST, #9664), PARP (CST, #9542), Cleaved PARP (Asp214) (CST, #5625), Histone H2A.X (Abcam, #ab11175), Phospho-Histone H2A.X (Ser139) (CST, #2577), P53 (DO-1) (Santa Cruz Biotechnology, #sc-126), CDK2 (78B2) (CST, #2546), phospho-CDK2 (Thr160) (CST, #2561), Rb (4H1) (CST, #9309), Phospho-Rb (Ser807/811) (CST, #8516), cdc2/CDK1 (POH1) (CST, #9116), and phospho-cdc2/CDK1 (Tyr15) (CST, #4539) at 1:1000; PLK1 (Santa Cruz Biotechnology, #SC-17783) and WEE1 (Santa Cruz Biotechnology, #SC-5285) at 1:200, and ß-actin (Sigma-Aldrich, #A2228) at 1:10000. The membranes were incubated with the primary antibodies overnight at 4 °C. Subsequently, the membranes were washed and incubated for 60 min at room temperature (RT) with the corresponding secondary antibodies, including anti-Mouse IgG HRP-linked (CST, #7076) and anti-Rabbit IgG HRP-linked (CST, #7074), at a dilution of 1:5000. Amersham ECL Select (Cytiva, Marlborough, MA, USA) and ImageQuant LAS 4000 (GE Healthcare Life Sciences, Piscataway, NJ, USA) were used for protein detection.

### Gene silencing

Small interfering RNAs (siRNAs) were transfected with Lipofectamine RNAiMAX Transfection Reagent (Invitrogen, Carlsbad, CA, USA) following the manufacturer’s protocol for RNAiMax reverse transfections. The sequences and sources of the siRNAs used in this study were as follows: siCCNE1#1 (custom from Merck), sense: 5′-GUAUAUGGCGACACAAGAA-3′ and antisense: 5′-UUCUUGUGUCGCCAUAUAC-3′; siCCNE#2 (Thermo Fisher Scientific, #4390824, s2524); siTP53#1 (custom from Merck), sense: 5′-GAAAUUUGCGUGUGGAGUAUU-3′ and antisense: 5′-AAUACUCCACACGCAAAUUUC-3′; siTP53#2 (custom from Merck), sense: 5′-GUGCAGCUGUGGGUUGAUUUU-3′ and antisense: 5′-AAAAUCAACCCACAGCUGCAC-3′. Negative control siRNA was purchased from Sigma-Aldrich (#SIC001). All siRNAs were used at a final concentration of 10 nM.

### Cell viability assay

Cells were treated with volasertib (PLK1 inhibitor; Selleckchem, Japan; S2235) or adavosertib (WEE1 inhibitor; Selleckchem, Japan; S1525) for 72 h. Alternatively, after siCCNE1 treatment for 48 h, cells were exposed to volasertib or adavosertib for an additional 72 h and cultured until further experimentation. Cell Count Kit-8 solution (FUJIFILM Wako; 341-07624) was added to each well, and cells were incubated for 2 h. The absorbance of the solution was measured at 450 nm using a microplate reader (BioTek, Winooski, VT, USA). Cells treated with dimethyl sulfoxide (DMSO; D2650, Sigma-Aldrich) were used as controls. The experiment was performed in triplicate.

### Cell cycle analysis and isobologram analysis

Cells treated with volasertib or adavosertib for 24 and 72 h were subsequently fixed with 70% ethanol and incubated overnight at 4 °C. RNase A stock solution (Sigma-Aldrich; R4875; final concentration: 0.5 mg/mL) was added, and cells were incubated for 20 min at 37 °C. Propidium iodide (PI, 50 mg/mL; Sigma-Aldrich; P4170) was added, and cells were incubated for 15 min at 4 °C in the dark. The proportion of cells in different phases of the cell cycle was measured via fluorescence-activated cell sorting (FACS) on a BD FACSCalibur™ HG Flow Cytometer Instrument (BD, Franklin Lakes, NJ, USA). The Cell Quest Pro software v3.1 (BD) and FlowJo® v10 (BD Biosciences, San Jose, CA, USA) were used for analysis. To distinguish between G2 and M phases in cells with 4N DNA content, dual staining was performed using Anti-Cyclin A2-FITC (Beckman Coulter, Brea, CA, USA; A22327) and 7-AAD (Beckman Coulter; A07704), following the protocol provided by Beckman Coulter (Beckman Coulter Cytometry Application Note 28). Cyclin A2 expression is low during early M phase, allowing for this distinction, as described in the protocol. Gating was performed by first identifying the 2N and 4N populations on the *x*-axis (7-AAD area) corresponding to the G1 and G2/M phases, respectively. The *y*-axis (Cyclin A2 area) was used to establish a threshold based on the Cyclin A2 expression level in G1 phase cells. Cells with 4N DNA content and Cyclin A2 expression above the threshold were gated as the G2 phase, while those below the threshold were gated as the M phase. Compensation was adjusted using IgG1 (mouse)-FITC Isotype Control (Beckman Coulter; A07795). Flow cytometry was performed using a CytoFLEX Flow Cytometer (Beckman Coulter) according to standard dual-color flow cytometry procedures. The experiment was repeated three times.

Combination effects of volasertib and adavosertib were assessed using combination index (C.Index) values and isobologram analysis. Cell viability was measured after 72 h of treatment. Isobologram analysis was performed to evaluate the interaction between volasertib and adavosertib. The IC_50_ values of each drug were calculated individually and in combination using cell viability assays. For the isobologram, the *x*-axis represents the IC_50_ of volasertib along with its 95% confidence interval (CI), while the *y*-axis represents the IC_50_ of adavosertib and its 95% CI. The isobologram was constructed by connecting the IC_50_ values and their CIs to define the region of additive effects. Points that fall above this region indicate antagonistic effects, while points below the region indicate synergistic effects.

### Cyclin E1 overexpression by doxycycline (Dox)-inducible system

Cyclin E1 overexpression was achieved using the PiggyBac transposon system. Cells were co-transfected with the PiggyBac plasmid pPB[TetOn]-Bsd-TRE > hCCNE1 (VectorBuilder, Chicago, IL, USA, Vector ID; VB241110-1370fjp), which encodes Cyclin E1 under the control of a doxycycline (Dox)-inducible promoter and a blasticidin resistance gene, and the helper plasmid pBase expression vector (VectorBuilder, Vector ID; VB10000-9365tax), which expresses the PiggyBac transposase. Transfection was performed using Lipofectamine™ 3000 reagent (Thermo Fisher Scientific, L3000015) and P3000™ Enhancer Reagent (Thermo Fisher Scientific, L3000015) in Opti-MEM™ Reduced Serum Medium (Thermo Fisher Scientific, 31985070), following the manufacturer’s instructions. After 24 h, the transfection medium was replaced with the complete medium supplemented with 10 μg/mL blasticidin S (Thermo Fisher Scientific, 461120). Cells were maintained under selection for 7 days to ensure the enrichment of successfully transfected cells. Cyclin E1 expression was induced by adding Dox (Doxycycline hyclate, Sigma-Aldrich, D9891) to the culture medium at a final concentration of 0.5 μg/mL. After 24 h of Dox treatment, cells were harvested to assess the efficiency of Cyclin E1 upregulation through qPCR and western blot analysis. To further evaluate the impact of Cyclin E1 overexpression, proliferation assays were performed in the presence of inhibitors.

### Time-lapse microscopy for apoptosis detection

Time-lapse microscopy was employed to monitor apoptosis dynamics in cells treated with inhibitors. In brief, cells were seeded into 12-well plates and allowed to adhere overnight. Following the addition of the WEE1 inhibitor, adavosertib, at 0.5 μM, CellEvent™ Caspase-3/7 Green ReadyProbes™ Reagent (Thermo Fisher Scientific, Catalog No. R37111) was immediately added to the culture medium at the recommended concentration to label apoptotic cells. The plate was placed into a live-cell imaging system (IncuCyte ZOOM, Essen Bioscience, Ann Arbor, MI, USA) in a humidified incubator at 37 °C with 5% CO_2_. Images were captured automatically every 2 h using a 20× objective lens in both phase contrast and green fluorescence channels to track apoptosis progression.

### Immunohistochemistry

The tumors were collected, and IHC staining was performed in xenograft models. The fixed tissue samples were dehydrated in graded ethanol, xylene, and embedded in paraffin. The paraffin blocks were cut into 3-μm sections, and each sample was subjected to hematoxylin and eosin (H&E) staining and IHC. For IHC, deparaffinized sections were blocked by incubation with 3% hydrogen peroxidase for 5 min at RT. Antigen retrieval was performed using an Antigen Unmasking Solution and citric acid-based (H-3300, Vector, CA, USA) for 10 min at 120 °C in a pressure cooker. Non-specific binding was blocked using ImmunoBlock (CTKN001, KAC, Amagasaki, Japan) for 5 min at RT. Sections were incubated overnight at 4 °C with primary antibodies against phospho-histone H2A.X (Ser139) (Cell Signaling Technology, #9718; 1:100) and Cyclin E1 (Proteintech, 11554-1-AP; 1:1000). After washing in TBS, the sections were treated with secondary antibody (Simple stain MAX-PO (R), 424142, Nichirei, Tokyo, Japan) for 30 min at 37 °C. To visualize the antigen-antibody complex, ImmPACT DAB substrate peroxidase (SK-4105, Vector Laboratories, Burlingame, CA, USA) was used, and the sections were counterstained with hematoxylin. Sections were observed using an Olympus BX53 microscope with a 20× objective lens, and images were captured using an Olympus DP74 camera (Olympus, Tokyo, Japan). Image acquisition and analysis were performed using the Olympus cellSens Standard 3.2.

### Ethics approval and consent to participate

Animal experiments were approved by the Institutional Animal Care and Use Committee of the University of Tokyo (Approval No.: A24M0450). All animal experiments were conducted in accordance with the relevant guidelines and regulations, including the ARRIVE guidelines and The University of Tokyo Animal Experimentation Manual (Japanese version). All in vitro experiments using cell lines were performed in accordance with institutional guidelines. The cell lines were purchased from established cell banks (such as ATCC and JCRB) and authenticated before use. This study did not involve human participants, and no specimens from cancer patients were included.

### Quantification and statistical analysis

Quantitative variables were analyzed using analysis of variance (ANOVA) (normally distributed data) and the Kruskal–Wallis H test (non-normally distributed data) for comparisons among the three groups. All reported *p* values were two-tailed, and *p* < 0.05 was considered significant (*p < 0.05, **p < 0.01, ***p < 0.001), unless otherwise specified. GraphPad Prism software 9.3.0 was used for all calculations, statistical analyses, and graphic representations.

## Supplementary information

Supplemental figures

## Data Availability

The datasets generated and/or analyzed during this study are available from the corresponding author. Any additional information required to reanalyze the data reported in this paper is available from the corresponding author upon request.
