# Supplementary material for: Cyclin E1 overexpression sensitizes ovarian cancer cells to WEE1 and PLK1 inhibition
Source: Oncogene. 2025 Feb 24;44(19):1375–86. doi: 10.1038/s41388-025-03312-4 (PMC12052589; doi:10.1038/s41388-025-03312-4)
Supplement: Supplementary file 1 — Supplemental figures [file 41388_2025_3312_MOESM1_ESM.docx]

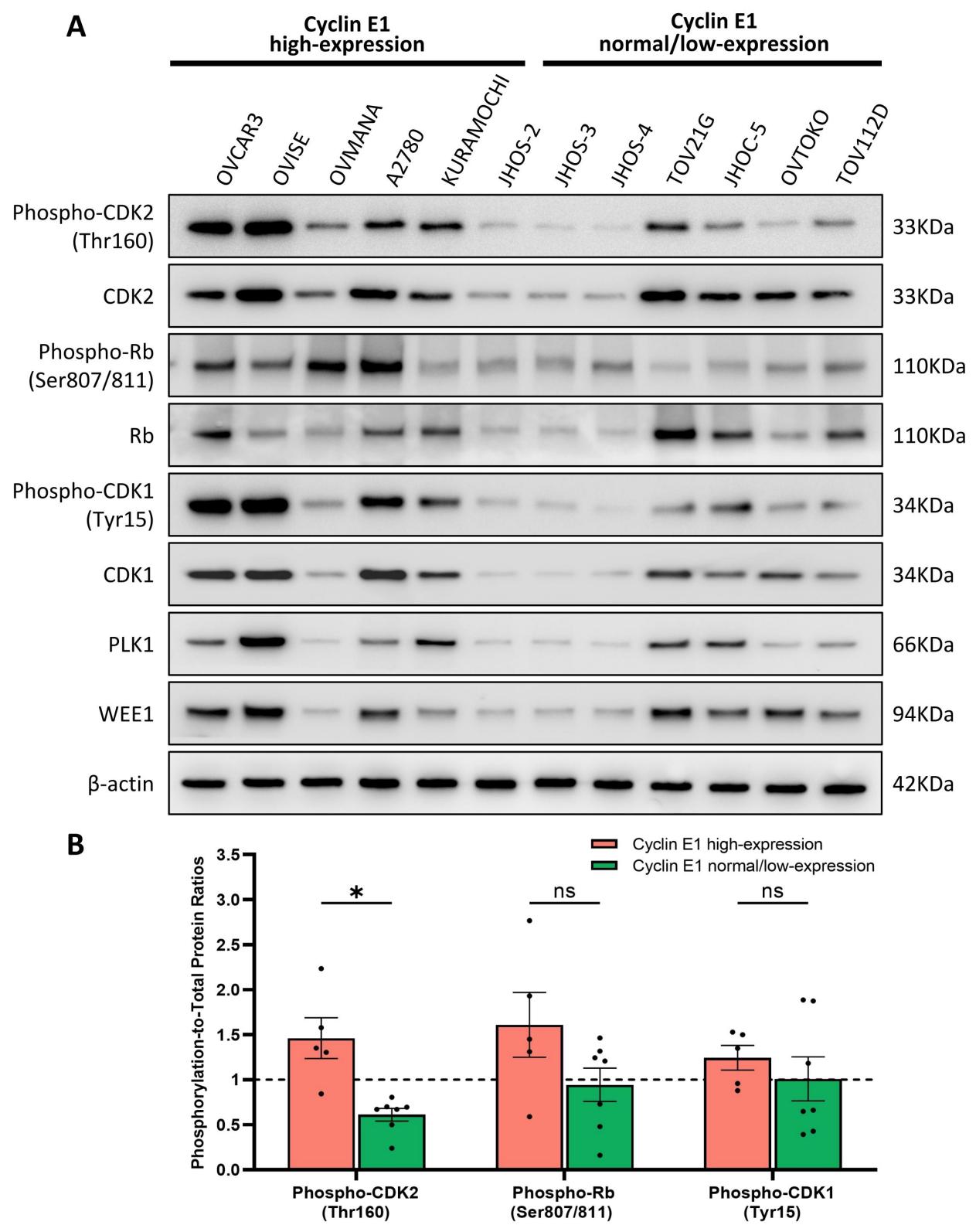


**Supplementary Figure 1. Phosphorylation levels of cell cycle checkpoint-related proteins in Cyclin E1-high and Cyclin E1-normal/low expressing cells**

Western blot analysis (A) and quantification (B) of phosphorylation levels of CDK2, Rb, and CDK1 in Cyclin E1-high and Cyclin E1-normal/low expressing cells. Quantification represents phosphorylation-to-total protein expression ratios, with data integrated from individual cell line results and shown as mean ± standard error of the mean (SEM). Statistical significance is indicated as **p* < 0.05 and ns (not significant).


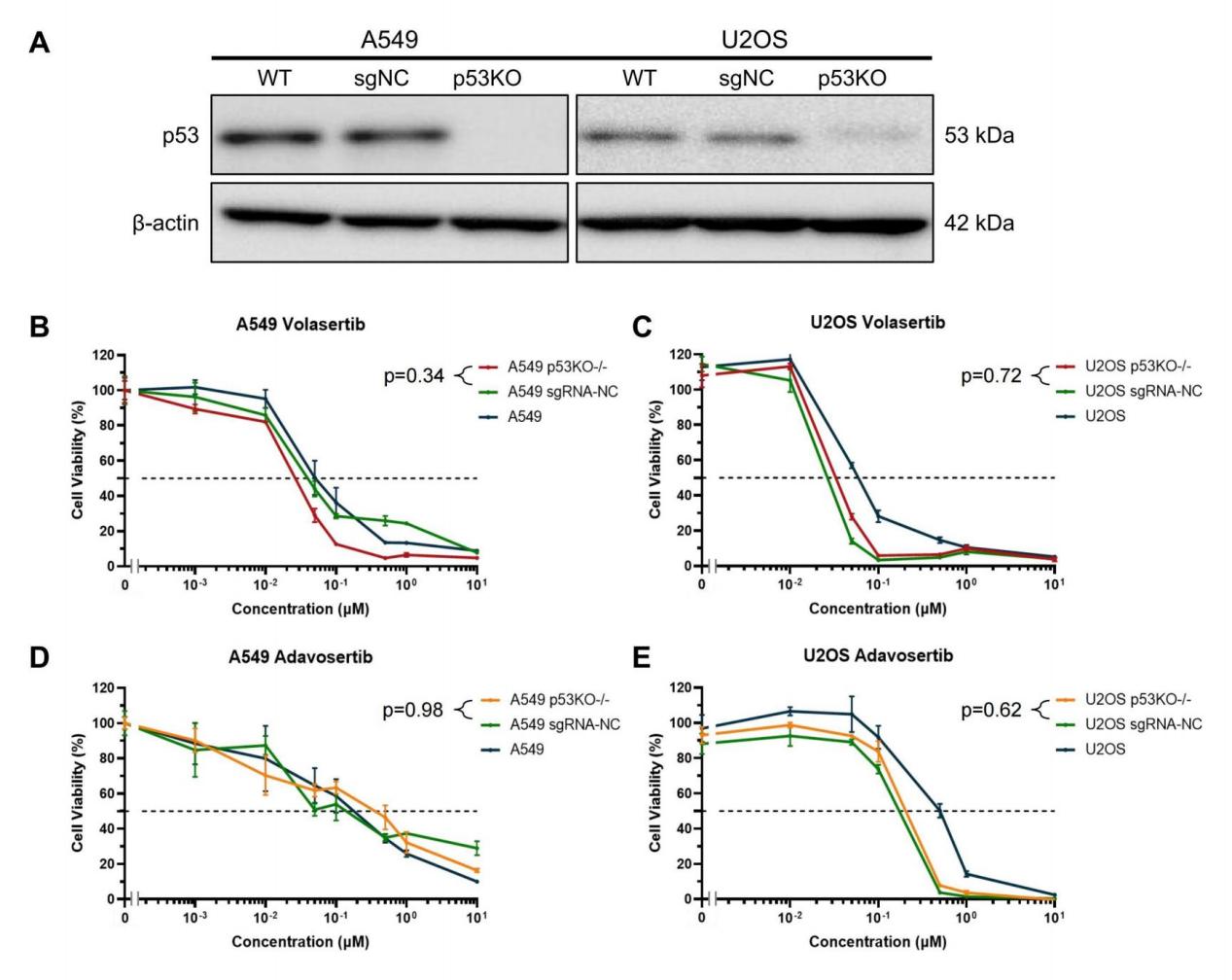


**Supplementary Figure 2. Drug sensitivity analysis for PLK1 inhibitor volasertib and WEE1 inhibitor adavosertib in *TP53* knockout (p53KO-/-) cell lines**

(A) Validation of the *TP53* knockout status using western blotting in p53KO-/- cells (A549: lung cancer, U2OS: osteosarcoma cell lines). sgNC: negative control sgRNA. (B-E) Analysis of cell viability in response to volasertib (B, C) and adavosertib (D, E) in parental, sgNC, and p53KO cells. Left: A549; Right: U2OS. The p-values were determined using a one-way ANOVA.


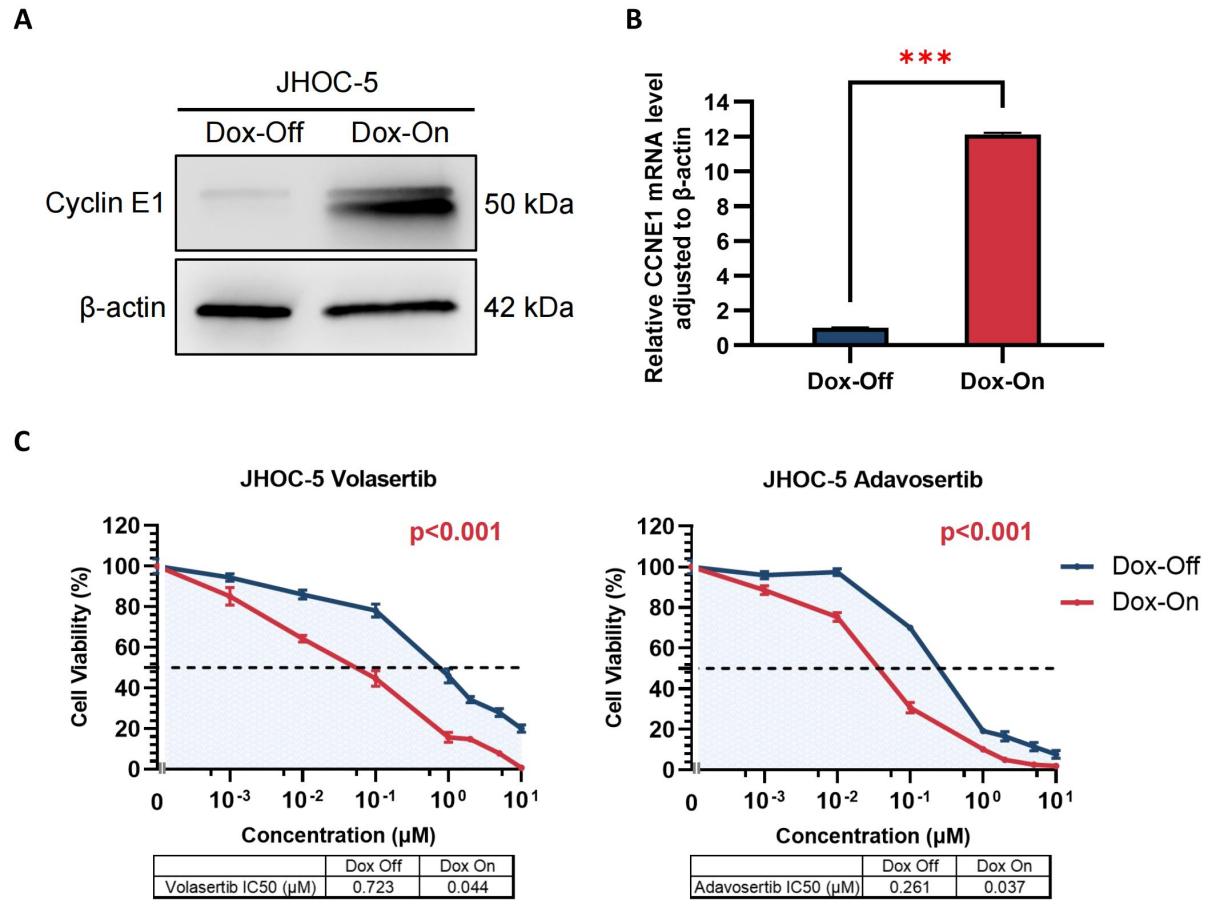


**Supplementary Figure 3. Drug sensitivity analysis for PLK1 inhibitor volasertib and WEE1 inhibitor adavosertib in Cyclin E1-upregulated cells using Dox-inducible system**

(A) Validation of Cyclin E1 upregulation at the protein level in JHOC-5 cells treated with doxycycline (Dox) (Dox On) or without doxycycline (Dox Off). (B) Quantification of Cyclin E1 mRNA levels by qPCR in Dox On and Dox Off conditions. (C) Analysis of cell viability in response to volasertib and adavosertib. Cyclin E1 upregulation was achieved by introducing a PiggyBac vector system and inducing expression with Dox. The p-values were determined using a t-test for qPCR and one-way ANOVA for cell viability assays. ****p* < 0.001.


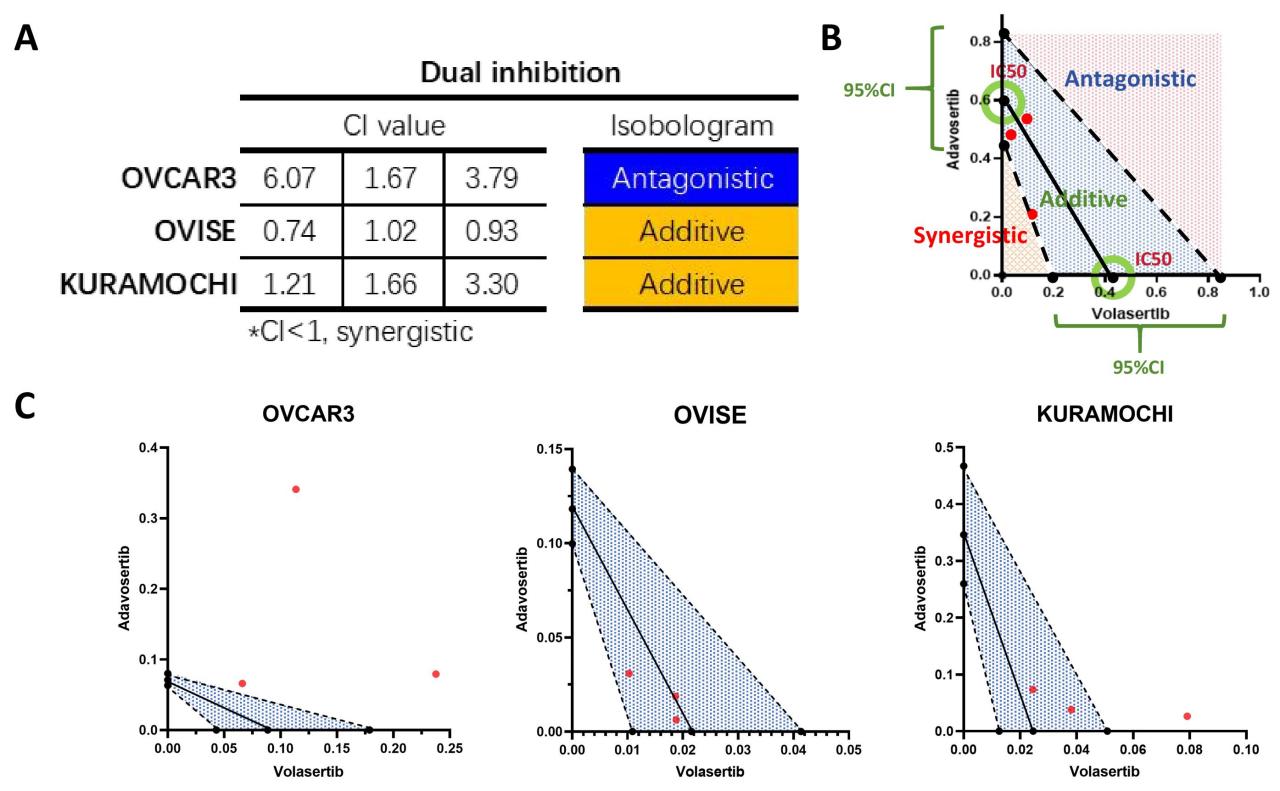


**Supplementary Figure 4. Combined effects of PLK1 and WEE1 inhibitors**

(A) Combination effects of volasertib (PLK1 inhibitor) and adavosertib (WEE1 inhibitor) were assessed using combination index (CI) values and isobologram analysis. (B) Isobologram explanation: The x-axis represents volasertib's IC50 and 95% CI, while the y-axis represents adavosertib's IC50 and 95% CI. The region formed by connecting these values indicates additive effects. IC50 values above the region indicate antagonistic effects, and those below indicate synergistic effects. (C) Isobologram results for OVCAR3, OVISE, and KURAMOCHI cell lines show the interaction of volasertib and adavosertib.
